# The 13^th^ World Congress for Neurorehabilitation, 22 - 25 May, Vancouver, Canada – *Advancing Neurorehabilitation across Time(s) and Continents*

**DOI:** 10.25122/jml-2024-1009

**Published:** 2024-06

**Authors:** Alexandra Gherman, Dafin Fior Muresanu

**Affiliations:** 1 RoNeuro Institute for Neurological Research and Diagnostic, Cluj-Napoca, Romania; 2 Department of Neuroscience, Iuliu Hatieganu University of Medicine and Pharmacy, Cluj-Napoca, Romania

**Figure 1:**
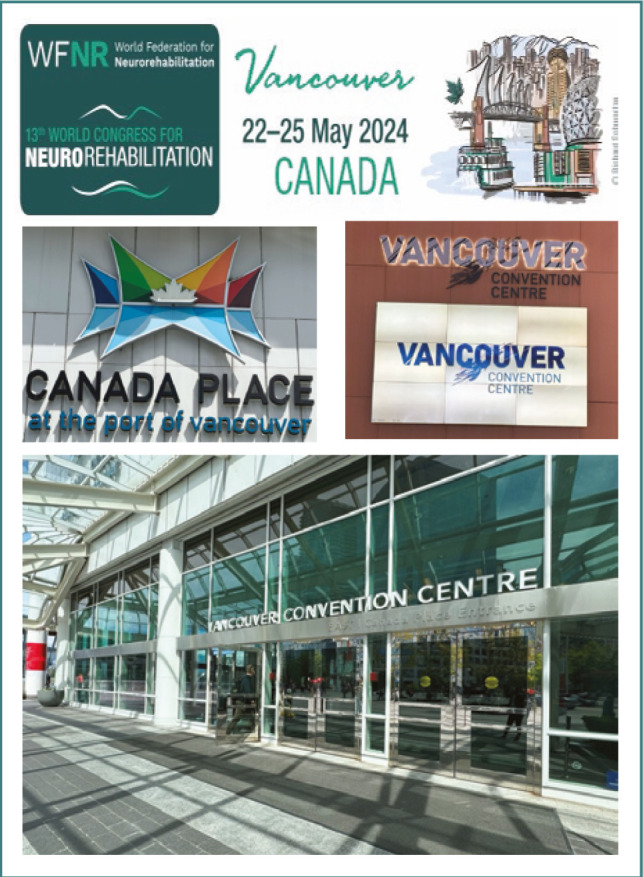
Canada Place and Vancouver Convention Centre – host of the 13^th^ World Congress for Neurorehabilitation (WCNR), Vancouver, Canada

Canada Place, Vancouver Convention Centre, East Building, May 22^nd^. At these iconic landmarks and venues (Figure 1), the World Federation for Neurorehabilitation welcomed more than 1.110 participants from over 50 countries, international world-renowned speakers, industry exhibitors and special guests to the 13^th^ edition of the World Congress for Neurorehabilitation, an event that spanned over four days (until May 25^th^). With a program containing more than 100 scientific sessions, be it plenary talks, symposia, round tables or workshops, the Congress again proved a milestone in the multidisciplinary landscape of neurorehabilitation, also allowing young specialists to bring forward their contributions via oral communications or poster presentations, and industry representatives to present their products to the audience in engaging discussions and debates. (*see Annex A for the 13^th^ WCNR in pictures*).

Annex A - The 13^th^ WCNR in pictures

**Figure 2:**
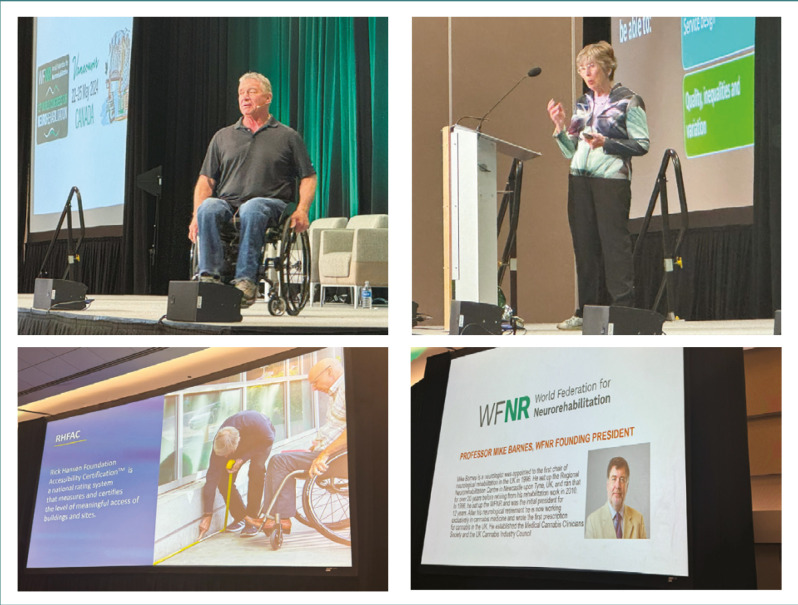
Rick Hansen and Prof. Pam Enderby – Opening Lectures at 13^th^ WCNR, Vancouver, Canada

The Opening Ceremony of the event started with the welcome of all participants addressed by the President of the World Federation for Neurorehabilitation, Prof. Dr Volker Hömberg, the congress chairs, Prof. Janice J. Eng and Dr Noah D. Silverberg (Vancouver, Canada), and the Chief Accessibility Officer of Canada, Stephanie Cadieux (Surrey, Canada), who all emphasised the role of neurorehabilitation at an international scientific level, the importance of collaboration among its various disciplines, and the future perspectives of neurorehabilitation, especially within the technological advancements frame. Their speeches were supported by two outstanding opening lectures delivered by Rick Hansen (Richmond, Canada) and Prof. Pam Enderby (Sheffield, UK) (Figure 2), with the first to engage the audience in a thrilling journey of the Man in Motion Tour (March 1985 – May 1989) just to outline how much courage, resilience, and strength lies within a person in the pursuit of quality of life, and the latter to deliver an emotional Barnes lecture, dedicated to Prof. Mike Barnes, WFNR Founding President, whose continuous efforts are directed towards raising the profile of neurorehabilitation as a discipline and enhancing the understanding and knowledge of the patient to ensure better outcomes, globally.

**Figure 3:**
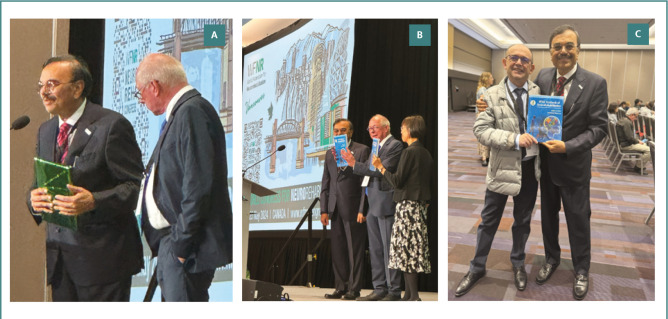
Announcing the launching of the IFNR Textbook on NeuroRehabilitation A) Prof. Dr Nirmal Surya (left) & Prof. Dr Volker Hömberg (right); B) Prof. Dr Nirmal Surya (left), Prof. Dr Volker Hömberg (centre) & Prof. Janice J. Eng (right); C) Prof. Dr Dafin Muresanu – President of the European Federation of Neurorehabilitation, WFNR Treasurer (left) & Prof. Dr Nirmal Surya

As a last point of the introduction, it is worth mentioning the surprise the audience was part of at the Opening Ceremony, i.e., the launching of the IFNR Textbook on Neurorehabilitation (Figure 3), announced by its editor-in-chief, Prof. Dr Nirmal Surya, President of the Indian Federation of Neurorehabilitation. The Textbook is an in-depth guide to all professionals working in the field of neurorehabilitation and it tackles topics such as the assessment of neurological deficits, implementation of evidence-based treatment strategies, and management of rehabilitation across different stages of recovery, with an emphasis on individual treatment plans, integration of emerging technologies and research findings.

**Figure 4:**
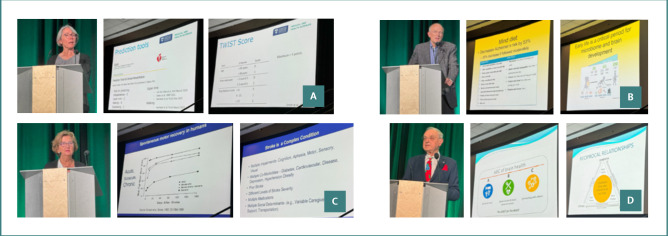
Photos from the plenary sessions: A) Prof. Cathy Stinear; B) Prof. Brett Finlay ; C) Prof. Pamela W. Duncan ; D) Prof. Vladimir Hachinski

Equally successful were the other four plenary sessions (Figure 4) contained in the program, offering the participants insights on:

- *Predicting Motor Outcomes: How and Why* by Prof. Cathy Stinear (Auckland, New Zealand);
- *The gut-brain axis: it’s all about the microbiome* by Prof. Brett Finlay (Vancouver, Canada);
- *Redesigning rehabilitation research for impactful and transformative outcomes: examples from stroke research* by Prof. Pamela W. Duncan (Seven Lakes, NC, USA);
- *Individual resilience in acute ischemic stroke: operationalizing brain reserve* by Prof. Dr Dafin Muresanu (Cluj-Napoca, Romania);
- *A critique on present and future of Robotic in Neurorehabilitation in LMIC* by Dr Rakesh Kumar Srivastava (New Delhi, India);
- *The Toronto Rehab TBI Recovery Study: from Bench to Bedside* by Dr Robin Green (Toronto, Canada)’
- *Stroke and dementia prevention: can neurorehabilitation do more?* by Prof. Vladimir Hachinski (London, Ontario, Canada).

**Figure 5:**
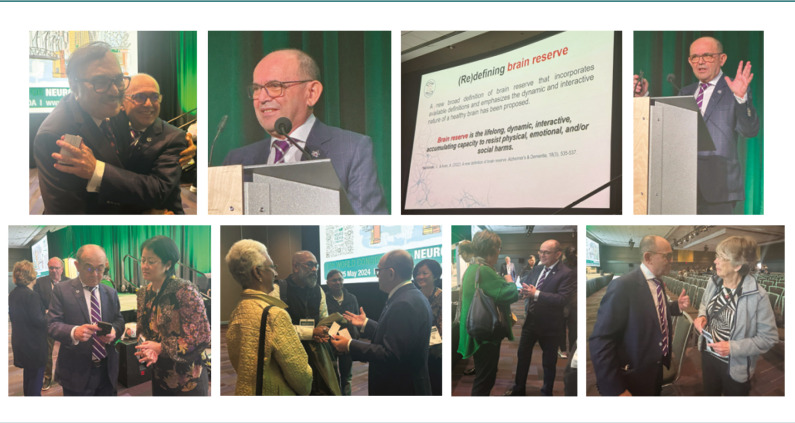
Prof. Dr Dafin Muresanu – photos from the plenary session and post-lecture discussions

Highly acclaimed by the audience and congratulated by peers was Professor D. Muresanu’s lecture on brain reserve (Figure 5), discussing the concept, its status quo, definition, theoretical and neurobiological basis, defining the neurocognitive domains and the neural networks, and outlining the dichotomy of the salience network and the central-executive network. Based on the interrelation between the brain’s level of organization, the top-down and bottom-up models of network functionality, and the potential of biomarkers to predict and influence outcomes, Prof. Dr Muresanu presents the development of the Individual Resilience Index (IRI), making an in-depth analysis of the indicators – structural, functional, metabolic and impact factors that were used for the piloting of IRI. A main takeaway from the lecture is that the IRI pilot can be used to inform future large-scale clinical trials to investigate neurobiological reserve in stroke.

“As a neurologist, I’m convinced that the concept of neurobiological reserve will become central to neurosciences in the coming decade. Biological reserve (BR), which reflects the brain’s capacity to compensate for and recover from neurological injury, is critical in shaping our approaches to patient care. By understanding and enhancing this reserve, we can develop more personalized and effective strategies for neurorehabilitation that not only promote recovery but also promote long-term outcomes. This focus on neurobiological reserve will drive innovations in clinical research, refining therapeutic interventions, and will be essential in the education of future neurologists. In my capacity as President of the European Federation for Neurorehabilitation Societies (EFNR), and based on the feedback received from congress participants, I strongly believe that BR is a game changer in the field of neurorehabilitation.”

*Prof. Dr. Dafin Muresanu*

*EFNR President*

This 2024 edition of the World Congress for Neurorehabilitation also constructed its successful substance on the multitude of workshops and seminars topics tackled, such as telerehabilitation, assessment and management of dysautonomia, Neurologic Music Therapy (NMT), GRASP (graded repetitive arm supplementary program), brain-computer interfaces, management of movement disorders, neuropathic pain and spasticity management after spinal cord injury (SCI), optimization of botulinum toxin A (BoNT-A) treatment, ultrasound-guided pain interventions, assessment and rehabilitation of visual perception and inattention difficulties, vestibular rehabilitation, family- and community-based rehabilitation after traumatic brain injury in developing and underdeveloped countries, as well as research methods and writing styles in neurorehabilitation journals, advances in therapeutic strategies for neural regeneration and neuroplasticity, transcranial direct current stimulation (tDCS), clinical applications of virtual reality (VR) to enhance post-rehabilitation outcomes, walking recovery after stroke, and implementation and clinical considerations in aquatic therapy for neurological rehabilitation; prehabilitation before neurosurgery, robotics and technology for rehabilitation, behavioral stroke biomarkers, challenges in pediatric neurorehabilitation, dysphagia, recent advances in the management of SMA (Spinal Muscular Atrophy), cannabis treatment for neurological conditions, wearable technology and robotics in neurorehabilitation, etc.. Noteworthy are also the adherent SIG (Special Interest Groups within the World Federation for Neurorehabilitation) meetings (e.g., on neuropharmacology, botulinum toxin, community-based neurorehabilitation, brain-computer interfaces, organization for psychological research into stroke, spinal cord injury, neuropathic pain, telerehabilitation, dementia) and symposia, each bringing forward the development of their specific area of interest (e.g., botulinum toxin, the management of invisible disability in Multiple Sclerosis (Multiples Sclerosis SIG WFNR & autonomic rehabilitation and cognitive neurorehabilitation SIG WFNR), the evaluation and implementation of PBS+PLUS (positive behaviour support intervention) in people with brain injury (neurorehabilitation SIG WFNR), as well as the joint symposia WFNR organized with, for example, the World Health Organization on the Intersectoral Global Action Plan implementation and brain health, the World Federation of Neurology (WFN), the American Academy of Neurology (AAN), as well as AOCNR (Southeast Asia and Oceania, on latest research on neurological rehabilitation), INDO AFRICAN (on funding and infrastructure challenges and solutions in the two regions), and ISPRM (on the significance of multimodal approach to spasticity and optimization of outcomes to patients).

**Figure 6:**
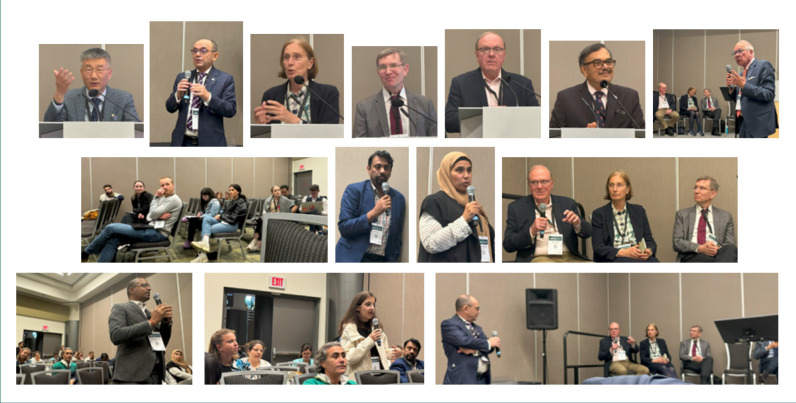
Photos of speakers and participants at the Career Development Round Table. From left to right: Dr Wayne Feng, Prof. D. Muresanu, Prof. S. Clarke, Prof. T. Platz, Prof. David Good, Prof. Nirmal Surya, Prof. V. Hömberg

Finally, a special section of the Congress program addressed the career development topic, bringing to a round table (Figure 6) career professionals like Dr Wayne Feng (Durham, NC, USA) who gave a thorough presentation of the WFNR mentor-mentee program, Prof. Dafin Muresanu (Cluj-Napoca, Romania) – session moderator, Prof. Dr Stephanie Clarke (Lausanne, Switzerland), Prof. Thomas Platz (Greifswald, Germany), Prof. David Good (Professor Emeritus at Penn State College of Medicine and the Past President of WFNR), Prof. Nirmal Surya (Mumbai, India) and Prof. Volker Hömberg (WFNR President), who all responded to questions addressed by the audience on the importance of leadership skills a rehabilitation professional should have to be established as an independent researcher, of mentorship programs and networking in career advancements, and also ways in which research and publication opportunities could be improved in the area of neurorehabilitation.

**Figure 7:**
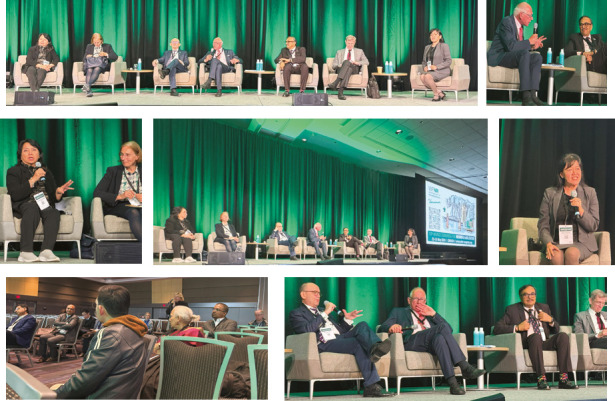
Presidential round table – Where do we go next? Center photo (form left to right) Prof. T. Joy Evangelista, Prof. S. Clarke, Prof. D. Muresanu, Prof. V. Hömberg, Prof. N. Surya (Mumbai, India), Prof. T. Platz (Greifswald, Germany), Assoc. Prof. M. Imamura

The presidential symposium *Where do we go next?* (Figure 7) approached the future trends of neurorehabilitation, targeting professional groups of neurologists, physiatrists, psychiatrists, psychologists and medical students. Prof. Volker Hömberg coordinated the discussions, engaging in dynamic dialogues with Assoc. Prof. Marta Imamura (University of São Paulo School of Medicine, São Paulo, Brazil), Prof. Stephanie Clarke (Lausanne, Switzerland), Prof. Teresita Joy Evangelista (University of the Philippines, the Philippines), Prof. Dafin Muresanu (Cluj-Napoca, Romania), Prof. Nirmal Surya (Mumbai, India), Prof. Thomas Platz (Greifswald, Germany), all of them outlining the utmost need to increase all efforts towards bringing together more effectively all specialists who are active in the field of neurorehabilitation and enhancing multidisciplinary approaches of collaboration and, as Marta Imamura pinpointed, the best way to have accomplishments is to engage in face-to-face interactions.

The 13^th^ Congress of the World Federation for Neurorehabilitation came to an end with the award ceremony of the 9^th^ Franz Gerstenbrand Award, which recognizes and rewards a neurorehabilitation project that has benefitted patients and went this year to Dr Cristian Endisch (Berlin, Germany) for his study *PREDICTING RECOVERY AFTER RESUSCITATION FROM CARDIAC ARREST* on the importance of the prognostics of SSEP recordings throughout the neurorehabilitation process from admission or at a later stage.

**Figure 8:**
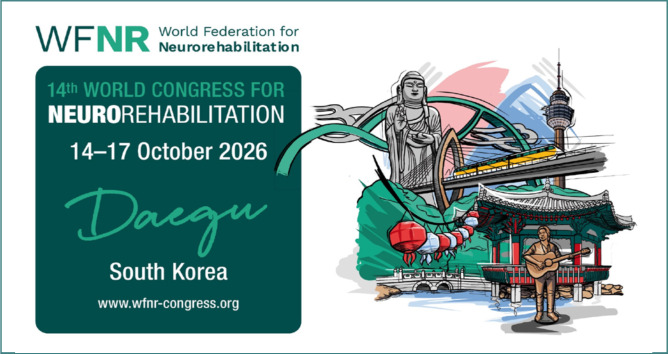
Poster of the 2026 WFNR Congress

Finally, here are some impressions recorded that best conclude the 2024 WCNR and make us look forward to the 2026 World Congress for Neurorehabilitation (Figure 8)!

“It has been a great Congress, with many people from 55 countries – that is great! Vancouver is a lovely city that I found out, it was a beautiful city to have the Congress in and it was a beautiful occasion with so many people, with so many different sessions to see, and so many interesting topics from one spectrum of rehabilitation to the other, it has been great! So, it has been lovely to be here and I look forward to coming back and seeing everyone again in South Korea!”

*Professor Mike Barnes*

*WFNR Founding President*

“We are just at the end of a very successful 13^th^ edition of the World Congress for Neurorehabilitation. I am always glad if the work is done but, nevertheless, let me briefly look back a little bit. I think we had a lot of highlights during the meeting and we, of course, have evolving trends in the field. One trend, certainly, is that we will concentrate much more than we have done before on community- and family-based aspects of rehabilitation; also, designing and refining communication tools for the education of relatives and caregivers, that is a very important point as well. The second point is that, fortunately, there is a growing interest in changing, in some sense, our way of thinking, and how we derive right decisions from knowledge. We have followed the concept of evidence-based medicine for decade now and, fortunately, there are emerging biometrical and epistemological ideas coming up, especially real-world data and similar aspects. Our pressure is, more and more, that the classical way of thinking in terms of evidence-based medicine is, probably, washing out many of the facts we could eventually gain from certain interventions. So, that is a second and certainly, important message from this meeting. We had a lot of very intriguing and interesting aspects in all fields of neurorehabilitation, the community of neurorehabilitation is growing and we will certainly, in the future, even try to have a higher political impact on what is going on. We have an increasing number of societies and members, and we will probably do more power playing in the political world, in the future, which also includes a new task force we founded for economic and political aspects, which is also necessary to do. So, that is a brief, meagre summary, you missed it to be here! You should think about coming in two years to Daegu, in South Korea! Thank you!”

*Professor Dr Volker Hömberg*

*President of the World Federation for Neurorehabilitation*

“This Congress was very splendid in terms of number of sessions, and quantity and quality. So, for the next Congress, we have a variety of specialities and interests, from basic home care to the very advanced AI applications in rehab, so that we can prove that we accept all kinds of things. What I want to place the focus on is that we want to embrace the young generation to have a chance to present their research so, I want to increase the oral sessions more broadly than ever – these are my thoughts. I would also like to say that Daegu is a unique place in Korean culture, so don’t hesitate to come to Daegu.”

*Professor Nam-Jonk Paik*

*President-elect of WFNR*

