# Supplementary material for: The 13th World Congress for Neurorehabilitation, 22 - 25 May, Vancouver, Canada – Advancing Neurorehabilitation across Time(s) and Continents
Source: J Med Life. 2024 Jun;17(6):543–8. doi: 10.25122/jml-2024-1009 (PMC11407490; doi:10.25122/jml-2024-1009)
Supplement: Supplementary file 1 [file JMedLife-17-543-s001.pdf]

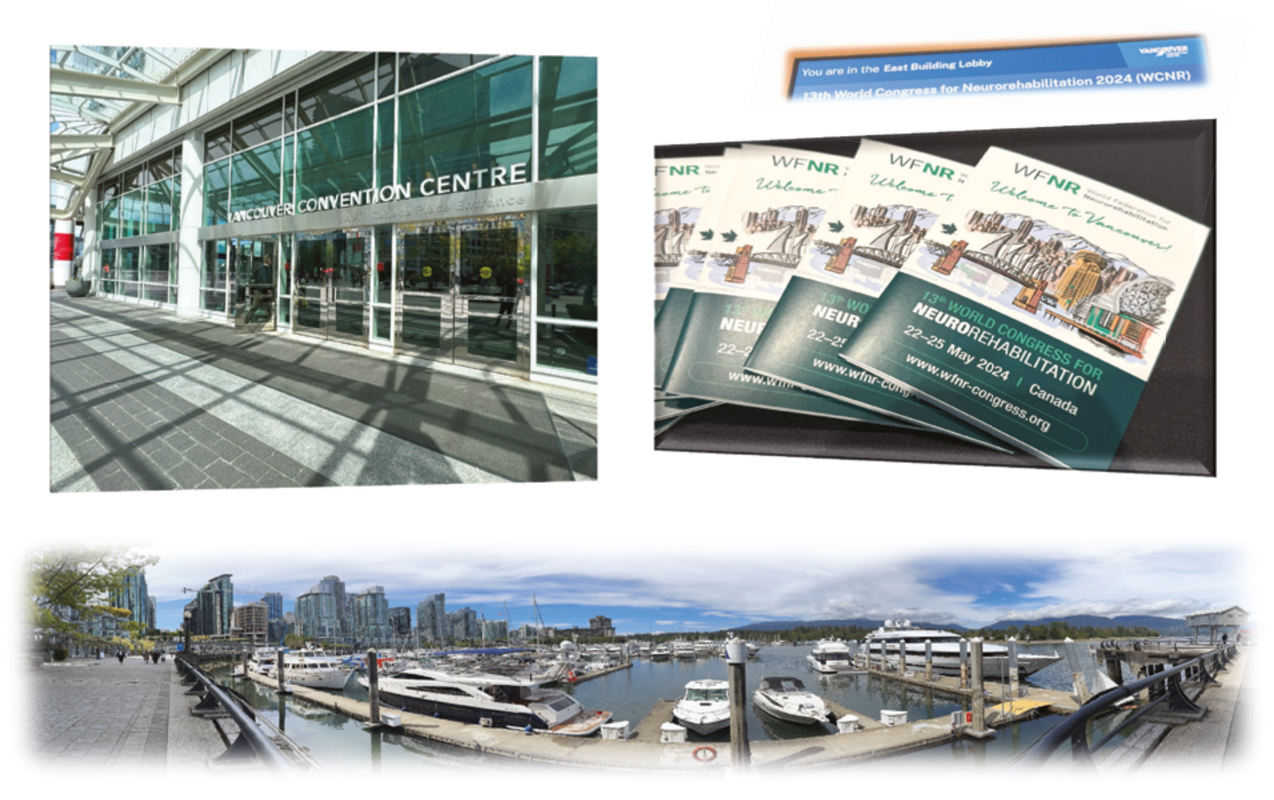

A. Vancouver Convention Centre – host of the 13th World Congress for Neurorehabilitation (WCNR), Vancouver, Canada

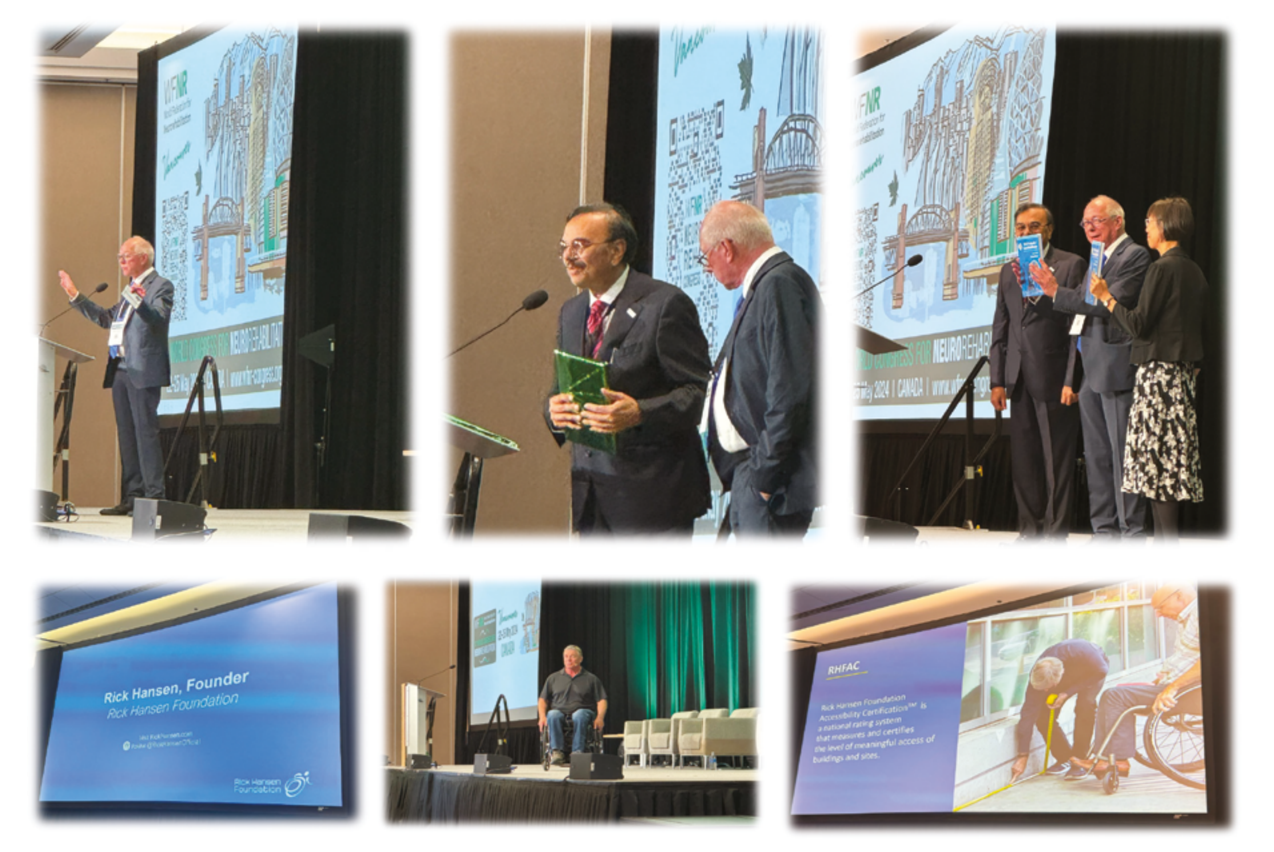

B. Opening Session of the 13th World Congress on Neurorehabilitation. Top row: Prof. Dr. Volker Hömberg – President of WFNR (left), with Prof. Dr. Nirmal Surya and his then-launched IFNR Textbook on NeuroRehabilitation (centre), and the two with Prof. Janice J. Eng (right). Bottom row: Opening lecture of Rick Hansen (centre).

© 2024 by the authors. This article is an open access article distributed under the terms and conditions of the Creative Commons Attribution (CC BY 4.0) license.

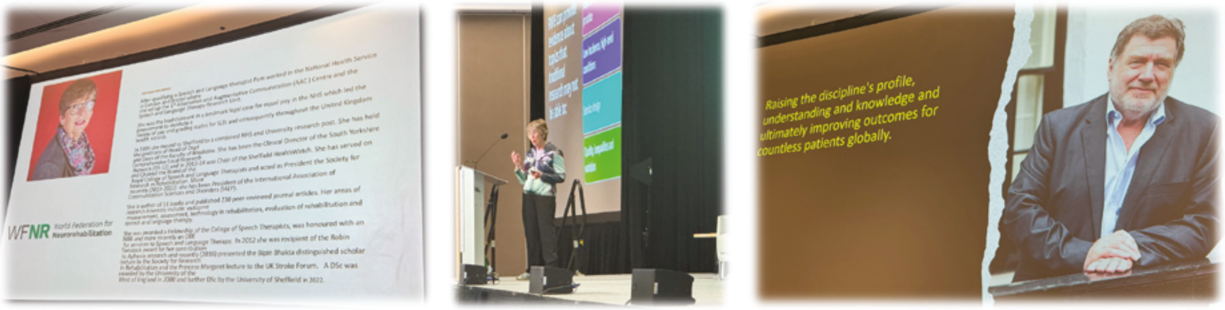

C. Prof. Pam Enderby - Opening Barnes lecture – dedicated to Prof. Dr. Michael Barnes, WFNR Founding President

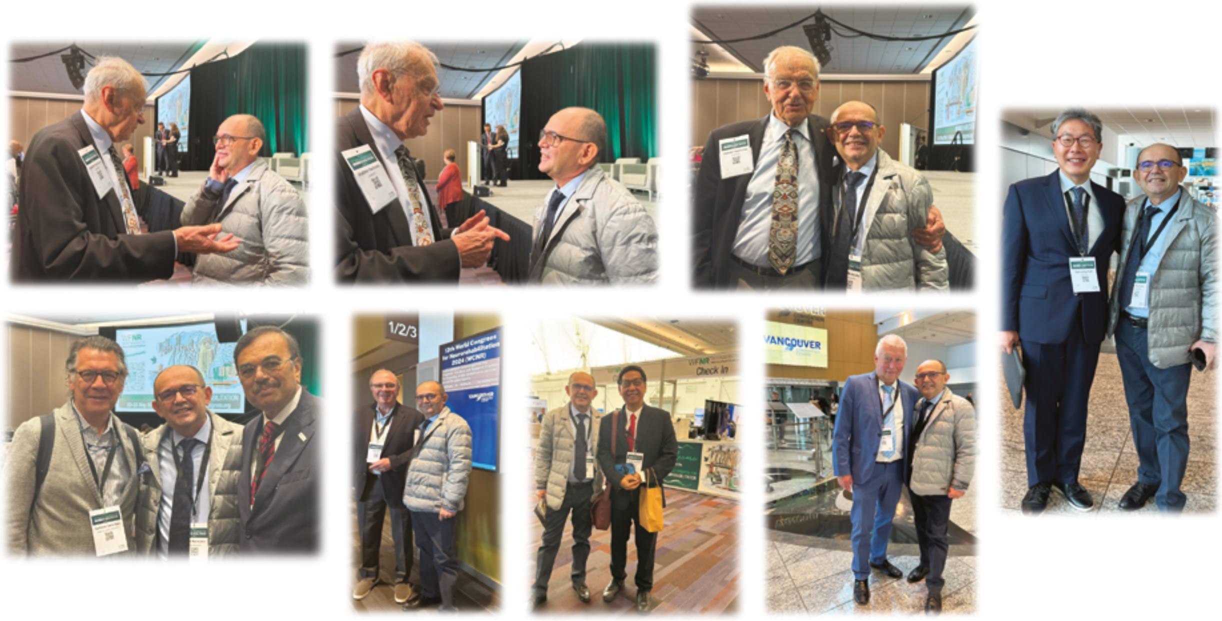

D. Prof. Dr. Dafin Muresanu – EFNR President and WFNR Treasurer & Vice President for the Eastern European Region with Prof. Dr. Vladimir Hachinski (top row) and (from left to right, bottom row) with Dr. Humberto Cerrel Bazo (Head of Spinal Cord Injury & Severe Acquired Brain Injury Unit, Treviso, Italy) & Prof. Dr. Nirmal Surya (President of the Indian Federation of Neurorehabilitation); with Prof. Dr. David Good (WFNR Past President), Dr. Reynaldo Reys-Matias (President of the Asian Oceanian Society of Physical and Rehabilitation Medicine); with Michael Taut (Professor of Music at the University of Toronto) & Prof. Dr. Nam-Jong Paik (President-Elect of WFNR).

© 2024 by the authors. This article is an open access article distributed under the terms and conditions of the Creative Commons Attribution (CC BY 4.0) license.

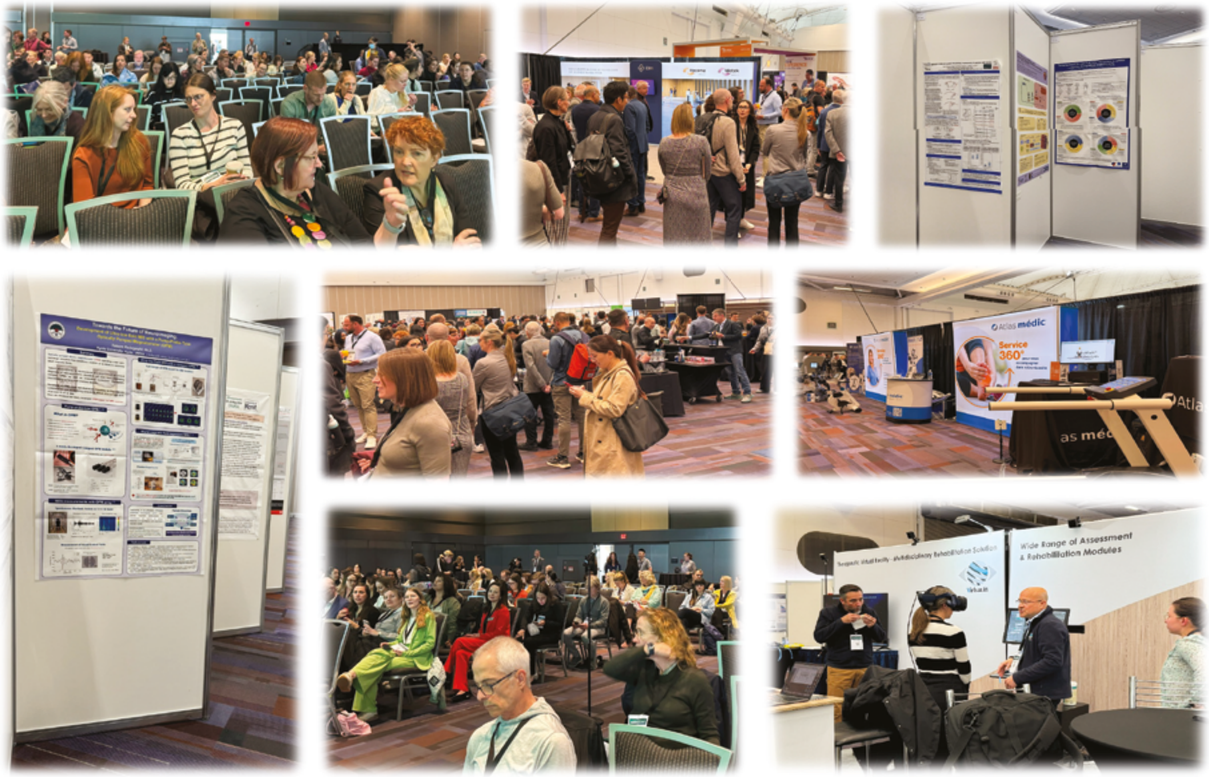

E. Participants, industry area and poster presentation area at the 13th WCNR

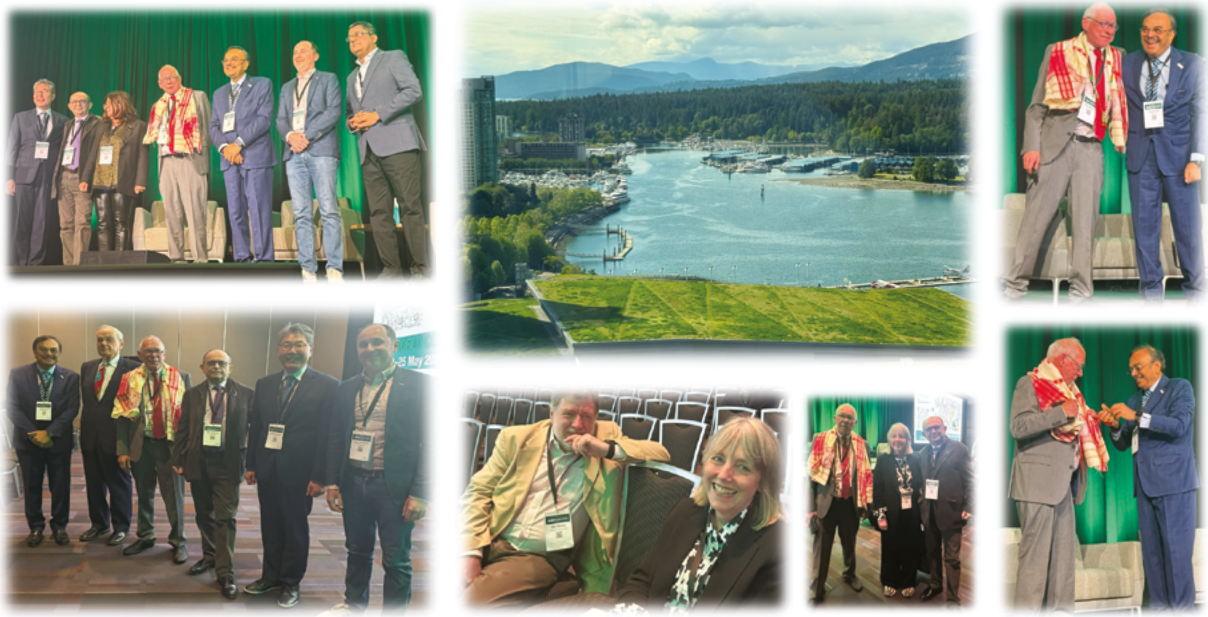

F. 13th WCNR Closing Ceremony, pictures and farewell

© 2024 by the authors. This article is an open access article distributed under the terms and conditions of the Creative Commons Attribution (CC BY 4.0) license.
